# The scavenging of superoxide radicals promotes apoptosis induced by a novel cell-permeable fusion protein, sTRAIL:FeSOD, in tumor necrosis factor-related apoptosis-inducing ligand-resistant leukemia cells

**DOI:** 10.1186/1741-7007-9-18

**Published:** 2011-03-19

**Authors:** Hongyun Tang, Yong Qin, Jianyong Li, Xingguo Gong

**Affiliations:** 1 Institute of Biochemistry, Zhejiang University, Hangzhou, 310058, PR China; 2 Institute of Biochemistry, College of Life Sciences, Zijingang campus, Room 345, Zhejiang University, Hangzhou, PR China

## Abstract

**Background:**

Many cancer cells develop resistance to tumor necrosis factor-related apoptosis-inducing ligand (TRAIL)-induced apoptosis, necessitating combination with chemotherapy, and normal cells manifest side effects due to the combined treatment regimen of TRAIL and chemotherapeutic drugs. A novel cancer therapy utilizing TRAIL is thus urgently needed.

**Results:**

In this study, we exploited TRAIL receptor-mediated endocytosis for the first time to produce a cell-permeable molecule, soluble forms of recombinant TRAIL:iron superoxide dismutase (sTRAIL:FeSOD), which possesses sTRAIL-induced apoptotic ability and FeSOD antioxidant activity. The FeSOD component was rapidly introduced into the cell by sTRAIL and intracellular superoxide radical (O_2_^-^), which have been implicated as potential modulators of apoptosis in cancer cells, was eliminated, resulting in a highly reduced cellular environment. The decrease in cellular O_2_^-^, which was accompanied by a brief accumulation of H_2_O_2 _and downregulation of phosphorylated Akt (p-Akt) and cellular FLICE-inhibitory protein, sensitized K562 leukemia cells and human promyelocytic leukemia (HL-60) cells to TRAIL-induced apoptosis. The low H_2_O_2 _levels protected human LO2 hepatocytes from sTRAIL:FeSOD-induced apoptosis despite downregulation of p-Akt. We also obtained evidence that the lack of response to sTRAIL:FeSOD in normal T cells occurred because sTRAIL:FeSOD shows much stronger shifts of redox state in erythroleukemia (K562) and HL-60 cells compared to that in normal T cells. K562 and HL-60 cells underwent sTRAIL:FeSOD-induced apoptosis without the dysfunction of mitochondria.

**Conclusions:**

The fusion protein overcomes the inability of FeSOD to permeate the cell membrane, exhibits synergistic apoptotic effects on K562 and HL-60 cells and demonstrates minimal toxicity to normal T cells and the normal liver cell line LO2, indicating its potential value for the treatment of leukemia.

## Background

Tumor necrosis factor-related apoptosis-inducing ligand (TRAIL) is a potent anticancer therapeutic agent that induces apoptotic cell death in cancer cells [1], regardless of P53 status. TRAIL is therefore a promising cancer therapeutic agent, especially for chemotherapy- or radiotherapy-resistant cancer cells [2]. Preclinical studies in mice and nonhuman primates with soluble forms of recombinant TRAIL (sTRAIL) have shown strong tumoricidal activity in xenografted tumor models without apparent toxic side effects [3,4]. However, certain TRAIL preparations have been shown to be toxic to human hepatocytes and keratinocytes, which may be responsible for the considerable hepatotoxicity or fulminant hepatic failure observed in human trials [5,6]. In addition, TRAIL resistance has been observed in many cancer cells [7-9]. Thus, understanding the exact molecular determinants of TRAIL resistance and developing strategies to overcome such resistance without killing normal cells are extremely important prerequisites for the successful deployment of TRAIL as a therapeutic agent.

Several different kinds of chemotherapy drugs are used in combination with TRAIL to sensitize TRAIL-resistant cancer cells, and many reports have combined recombinant TRAIL with standard anticancer therapies to induce synergistic tumor cell apoptosis [10,11]. However, there is evidence that some normal human cells are sensitive to apoptosis after treatment by TRAIL in combination with chemotherapeutic drugs [12,13]. Furthermore, mutation or deletion of *p53 *occurs in more than half of all human tumors, and Akt is frequently hyperactive in cancer cells. Both of these alterations play a prominent role in cell resistance to chemoradiotherapy. Edwin *et al. *[14] reported a recombinant fusion protein, single-chain variable fragment 425 (scFv425):sTRAIL, that combined the tumoricidal effect of epidermal growth factor receptor signal inhibition with target cell-restricted apoptosis induction, hence showing promising antitumor activity. Thus, in recent years, biological mechanism-based cancer therapeutic strategies that may exert enhanced antitumor activity and high tumor specificity have attracted much more attention because of the unfavorable side effects of chemoradiotherapy and the resistance of many tumor cells to chemo- or radiotherapy [2,15].

Antioxidants have long been used for the treatment of cancer, especially in combination with other anticancer drugs [16]. Superoxide dismutase (SOD) is a type of potent antioxidant enzyme that suppresses the growth of various cancer cells by removing superoxide radicals (O_2_^-^) [17], which are critical in different stages of carcinogenesis. However, owing to its large molecular weight, SOD cannot enter the cell to exert its effects. To overcome this deficiency, a liposome can be used to enclose SOD, allowing it to enter cells [18]. Additionally, we have previously shown that a fusion of SOD with scFv was able to permeate the cell membrane via receptor-mediated endocytosis and was able to then inhibit cell proliferation through the Akt/p27^kip1 ^pathway [19]. However, neither of these approaches effectively inhibits cancer cell proliferation, and therefore engineering SOD to permeate the cell membrane and exercise its powerful cytotoxic effects is key to its clinical application.

Akt regulates the transactivation of antiapoptotic molecules such as cellular FLICE-inhibitory protein (c-FLIP_L_), X-linked inhibitor of apoptosis protein and the antiapoptotic protein B-cell lymphoma-extra large (Bcl-x_L_) [20-22]. Furthermore, Akt is dephosphorylated at Thr308 (converting it into the inactive form) at reduced levels of intracellular reactive oxygen species (ROS) [23], making SOD an attractive therapeutic agent for sensitizing cancer cells to TRAIL-induced apoptosis. Because TRAIL can be internalized via receptor-mediated endocytosis [24], we hypothesized that iron superoxide dismutase (FeSOD) could be internalized with sTRAIL to reduce the level of intracellular O_2_^-^. Here we cloned and coexpressed sTRAIL (114 to 281 aa) and FeSOD, as well as a fusion protein containing both, to determine whether the resultant fused protein possessed dual activity and could exert a synergistic effect on cancer cells. We observed sTRAIL:FeSOD to engage the TRAIL receptors (TRAIL-R1 and TRAIL-R2), resulting in internalization of the receptor and ligand. Once inside the cell, sTRAIL:FeSOD scavenged intracellular O_2_^-^, which then influenced the TRAIL-induced apoptosis pathway.

## Results

### Production of sTRAIL:FeSOD

**Figure 1:**
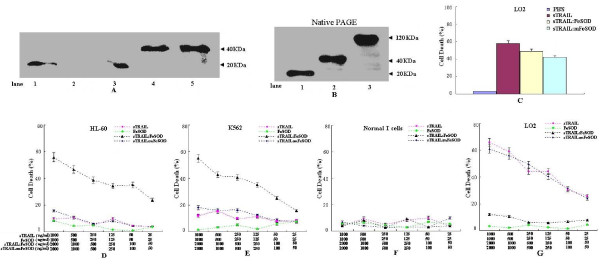
**Western blot analysis of soluble forms of recombinant tumor necrosis factor-related apoptosis-inducing ligand (sTRAIL), enzymatic activity deficient form of fused protein (sTRAIL:mFeSOD) and sTRAIL:iron superoxide dismutase (FeSOD) expression and sTRAIL:FeSOD-induced cell death**. **(A) **After isopropyl-β-D-thiogalactoside induction, the expressed sTRAIL, sTRAIL:mFeSOD or sTRAIL:FeSOD was subjected to Western blot analysis with anti-TRAIL antibody. Lanes 1 to 5 are sTRAIL control (Peprotech, Rocky Hill, New Jersey, USA) and extracts of BL-21 transformed with pET28, pET28-sTRAIL, pET28-sTRAIL:mFeSOD and pET28-sTRAIL:FeSOD, respectively. **(B) **Samples were prepared and electrophoresed using native polyacrylamide gel electrophoresis (PAGE) under nondenaturing conditions followed by electrotransfer and immunoblotting with TRAIL antibody. Lanes 1 to 3 are sTRAIL control (purchased from Peprotech), sTRAIL:FeSOD (before renaturation) and sTRAIL:FeSOD (renatured), respectively. **(C) **Six-well plates were seeded with LO2 cells and allowed to adhere before replacement with RPMI 1640 medium with hyperosmotic sucrose (0.25 M) containing sTRAIL (500 ng/ml), sTRAIL:FeSOD (1,000 ng/ml) or sTRAIL:mFeSOD (1,000 ng/ml). After treatment for 8 hours, cell death was quantified by flow cytometry. **(D) **through **(G) **Cells were grown in six-well plates to 60% confluence, and the indicated proteins were added at the indicated concentrations. After treatment for 8 hours, cells were stained with fluorescein isothiocyanate anti-annexin V antibody and propidium iodide and then examined using flow cytometric analysis. For each sample, 10,000 events were acquired. Results are expressed as the mean fluorescence intensity. Each bar represents the mean ± SE obtained from three independent experiments.

**Figure 2:**
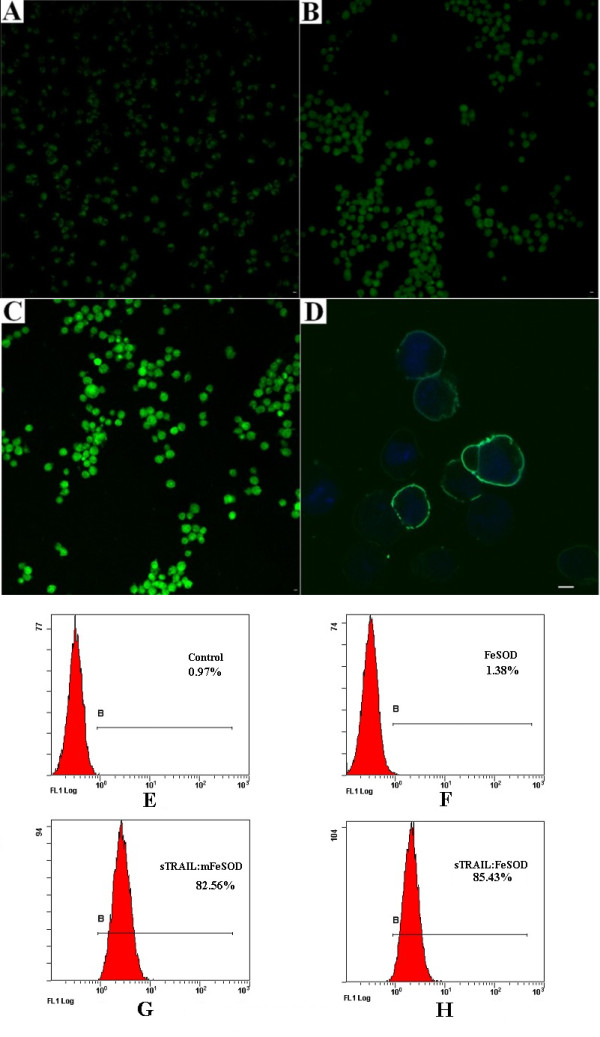
**Internalization of soluble forms of recombinant tumor necrosis factor-related apoptosis-inducing ligand:iron superoxide dismutase (sTRAIL:FeSOD) is rapid and is inhibited by hyperosmotic sucrose**. **(A) **through **(C) **Suspended erythroleukemia (K562) cells were treated with fluorescein isothiocyanate (FITC)-labeled sTRAIL:FeSOD (green) in RPMI 1640 medium without fetal calf serum for **(A) **5 minutes, **(B) **15 minutes or **(C) **30 minutes. After acid washes, cells were visualized with laser scanning confocal microscopy (LSCM). The LSCM parameters were set to an excitation wavelength of 488 nm, an emission wavelength of 500 to 550 nm and pinhole of 202 μm. **(D) **LO2 cells were preincubated for 30 minutes in the presence of 0.25 M sucrose at 37°C before incubation in the presence of FITC-labeled sTRAIL:FeSOD (green) for an additional 30 minutes. After being washed extensively in ice-cold phosphate-buffered saline (PBS), cells were counterstained with Hoechst 33342 (blue) and analyzed by LSCM. The white bar represents 5 μm. The LSCM parameters for FITC were set to an excitation wavelength of 488 nm, an emission wavelength of 500 to 550 nm and pinhole of 215 μm. For Hoechst 33342, the parameters were set to an excitation wavelength of 350 nm, an emission wavelength of 460 to 480 nm and pinhole of 215 μm. **(E-H) **After K562 cells were treated with PBS, FITC-labeled FeSOD, sTRAIL:FeSOD or sTRAIL:mFeSOD for 30 minutes, the internalization rate was quantified by flow cytometry.

After addition of isopropyl-β-D-thiogalactoside (IPTG), the expected proteins sTRAIL (21.6 kDa), enzymatic activity deficient form of fused protein (sTRAIL:mFeSOD) (42.6 kDa) and sTRAIL:FeSOD (42.6 kDa) were recovered as demonstrated by Western blot analysis with an anti-TRAIL antibody (Figure 1A). The sTRAIL:FeSOD and sTRAIL:mFeSOD proteins were expressed in inclusion bodies and required refolding to regain activity. The trimeric structure is the basis of sTRAIL-induced apoptotic function. To determine whether the FeSOD domain changes the oligomerization state of sTRAIL, renatured sTRAIL:FeSOD was collected and run over a Sephadex G-100 column (GE Healthcare, Fairfield, Connecticut, USA). The renatured proteins were identified to be homogeneous (data not shown). Renatured sTRAIL:FeSOD was further shown to be trimeric by performing native polyacrylamide gel electrophoresis (PAGE) Western blot analysis (Figure 1B). SOD activity assays indicated that the antioxidant activity of renatured sTRAIL:FeSOD was 2,300 U/mg, whereas that of natural *Nostoc commune *FeSOD was 3,500 U/mg. sTRAIL:mFeSOD show no antioxidant activity. The induction of apoptosis in TRAIL-sensitive LO2 cells indicated sTRAIL activity. To exclude the effects of FeSOD, the LO2 cells were incubated in RPMI 1640 medium with 0.25 M sucrose, a condition in which internalization was inhibited (Figure 2D) [25]. The sTRAIL activity assay results are shown in Figure 1C. LO2 cells treated in hyperosmotic medium with 500 ng/ml sTRAIL showed a 57.2% cell death rate, whereas sTRAIL:FeSOD and sTRAIL:mFeSOD treated with 1,000 ng/ml sTRAIL had cell death rates of 48.3% and 41.5%, respectively.

### Sensitization of K562 and HL-60 cell lines to sTRAIL:FeSOD

As described above, many tumor cells are resistant to TRAIL because of phosphorylated Akt (p-Akt) upregulation. Recently, we discovered that FeSOD can downregulate the levels of p-Akt [19], and therefore we were interested in determining whether FeSOD could cooperate with TRAIL to enhance the killing of tumor cells. To address this question, the TRAIL-resistant erythroleukemia (K562) cells and human promyelocytic leukemia (HL-60) cells [26] were used to detect the antitumor ability of sTRAIL:FeSOD. Compared with sTRAIL:mFeSOD or sTRAIL, K562 and HL-60 cells demonstrated significantly less viability after treatment with sTRAIL:FeSOD, and their antitumor activity increased significantly in a sTRAIL:FeSOD dose-dependent manner. The decrease in cell viability was ascribed to apoptosis as demonstrated by anti-annexin V antibody and propidium iodide (PI) staining (Figures 1D and 1E), strongly suggesting that sTRAIL and FeSOD synergistically induced apoptosis in the TRAIL-resistant K562 and HL-60 tumor cells. In contrast to the effect in these two cell lines, sTRAIL:FeSOD did not sensitize freshly isolated peripheral blood T cells to death induced by apoptosis (Figure 1F). As TRAIL induces apoptosis in normal human hepatocytes, the LO2 cells were also treated as a control group. Interestingly, the LO2 cells, which are extremely sensitive to TRAIL-induced apoptosis [5], were less sensitive to sTRAIL:FeSOD (Figure 1G).

### Rapid internalization of sTRAIL:FeSOD

The ability of sTRAIL:FeSOD to enter the cell is the basis for the synergistic effect of sTRAIL and FeSOD. After incubation with labeled sTRAIL:FeSOD for 5 minutes, the K562 cells began to show fluorescence (Figure 2A). Internalization increased at 15 minutes (Figure 2B) with marked fluorescence and fluorescence was even stronger by 30 minutes (Figure 2C). As the cell surface-associated ligand was stripped by acid washing, the internal fluorescence of the cells could be determined. Uptake of sTRAIL:FeSOD was completely blocked by hypertonic medium in LO2 cells (Figure 2D). Quantitative data detected by flow cytometry indicated internalization rates for FeSOD, sTRAIL:mFeSOD and sTRAIL:FeSOD of 1.38%, 82.56% and 85.43%, respectively (Figures 2E to 2H). Internalization of sTRAIL:FeSOD in HL-60 and LO2 cells, which underwent the same treatment processes as did the K562 cells, were confirmed by LSCM. However, normal T cells showed low fluorescence signals, which may be related to the minimal expression of TRAIL death receptors [27] (data not shown).

### sTRAIL:FeSOD neutralizes free radical O_2_^- ^and reduces ROS

**Figure 3:**
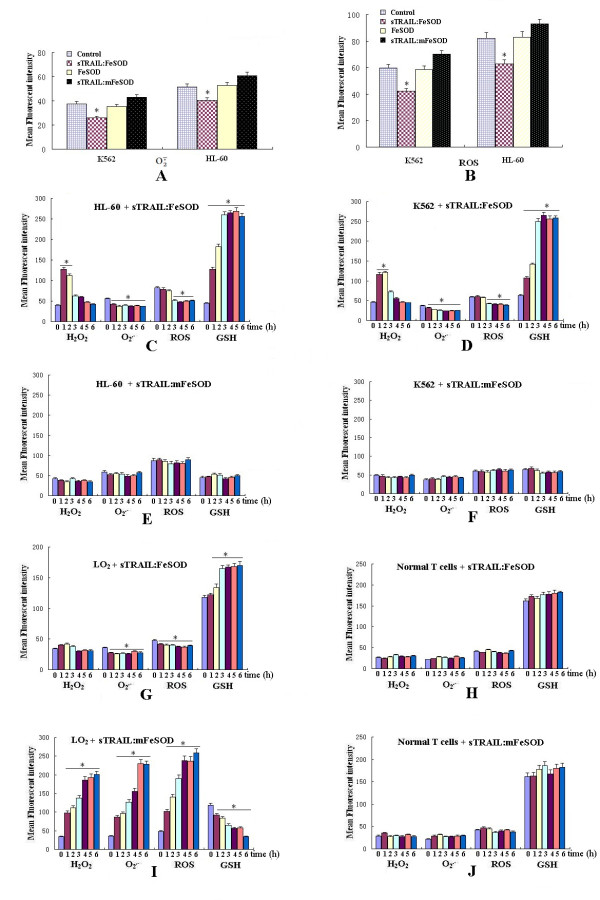
**Effects of soluble forms of recombinant tumor necrosis factor-related apoptosis-inducing ligand:iron superoxide dismutase (sTRAIL:FeSOD) on intracellular H**_**2**_**O**_**2**_**, glutathione (GSH), superoxide radical (O**_**2**_^-^**) and reactive oxygen species (ROS) levels**. **(A) **and **(B) **After treatment with sTRAIL:FeSOD (1,000 ng/ml), sTRAIL:mFeSOD (1,000 ng/ml) or FeSOD (500 ng/ml) for 3 hours, dihydroethidium (DHE) or dichlorodihydrofluorescein diacetate (DCFDA) was added for detection. **(C) **through **(J) **After cells were treated with sTRAIL:FeSOD (1,000 ng/ml) or sTRAIL:mFeSOD (1,000 ng/ml) for 0, 1, 2, 3, 4, 5 or 6 hours, intracellular H_2_O_2_, GSH, O_2_^- ^and ROS were measured by DHR123, NDA, DHE and DCFDA, respectively. The fluorescence was measured by flow cytometry. For each sample, 10,000 events were acquired. Results are expressed as the mean fluorescence intensity. Each bar represents the mean ± SE obtained from three independent experiments (*P < 0.05 vs. untreated control).

SOD is a potent antiradical enzyme, and Akt activity can be downregulated under low levels of ROS. To investigate the intracellular antioxidant activity of sTRAIL:FeSOD, we measured ROS levels using the oxidation-sensitive fluorescent dyes dichlorodihydrofluorescein diacetate (DCFDA) (for total ROS) and dihydroethidium (DHE) (for O_2_^-^). The O_2_^- ^and ROS levels in leukemia cells decreased significantly after pretreatment with sTRAIL:FeSOD (Figures 3A and 3B). However, in contrast to the phosphate-buffered saline (PBS) control, pretreatment with sTRAIL:mFeSOD or FeSOD did not cause a significant difference in these roles, ruling out a role for sTRAIL in O_2_^- ^quenching and confirming the low permeability of SOD.

SOD is well known to induce H_2_O_2 _production during O_2_^- ^scavenging, and therefore we monitored the changes in H_2_O_2_, O_2_^-^, ROS and glutathione (GSH) levels after treating the cells with sTRAIL:FeSOD. As shown in Figures 3C to 3F, H_2_O_2 _levels increased 1 or 2 hours after administration of sTRAIL:FeSOD in K562 and HL-60 cells in comparison with the levels in LO2 and T cells. However, at 3 hours after treatment, we observed that H_2_O_2 _levels had declined almost to normal levels. As compared with sTRAIL:mFeSOD, sTRAIL:FeSOD induced a notably more significant decrease in cellular ROS and O_2_^- ^fluorescence intensities and a marked rise in GSH levels in the K562 and HL-60 cells (Figures 3C to 3F). Figure 3G shows that the redox status in LO2 cells changed, but not as strongly as they did in the cancer cells or in the sTRAIL:mFeSOD-treated LO2 cells. As shown in Figure 3H, normal T cells maintained a stable redox status, which may explain why they did not undergo sTRAIL:FeSOD-induced apoptosis. Figure 3I shows that sTRAIL:mFeSOD-induced apoptosis in LO2 cells was accompanied by a rapid increase in ROS levels. The graph also shows that 3 hours after sTRAIL:FeSOD treatment in the two leukemia cell lines, ROS levels did not increase along with the decrease in H_2_O_2_, suggesting that H_2_O_2 _was broken down by the cellular antioxidant defenses rather than giving rise to highly reactive hydroxyl radicals [28].

### sTRAIL:FeSOD-induced apoptosis does not involve the mitochondrial apoptotic pathway

**Figure 4:**
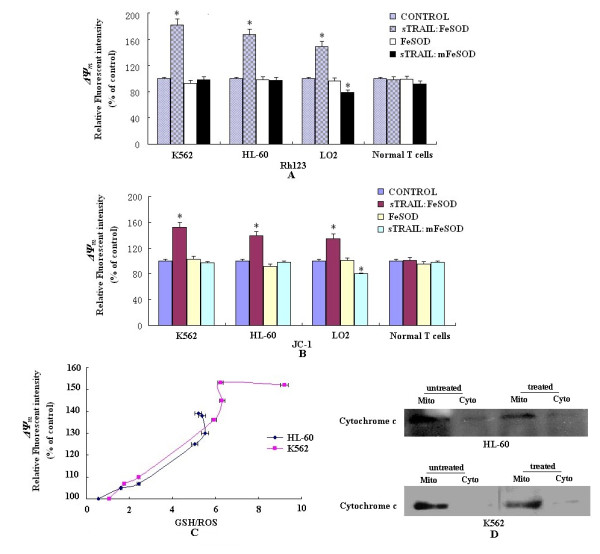
**Effects of soluble forms of recombinant tumor necrosis factor-related apoptosis-inducing ligand:iron superoxide dismutase (sTRAIL:FeSOD) on mitochondrial membrane potential (ΔΨm)**. **(A) **and **(B) **ΔΨm was measured with **(A) **rhodamine123 (Rh123) and **(B) **JC-1. After treatment with sTRAIL:FeSOD (1,000 ng/ml), sTRAIL:mFeSOD (1,000 ng/ml) or FeSOD (500 ng/ml) for 6 hours, cells were incubated for 30 minutes at 37°C with 10 μM Rh123 or 1 μg/ml JC-1 in phosphate-buffered saline and then subjected to flow cytometric analysis. For each sample, 10,000 events were acquired. Results are expressed as a ratio of the relative fluorescence intensity. Each bar represents the mean ± SE obtained from three independent experiments (*P < 0.05 vs. untreated control). **(C) **Kinetic relationship of redox state (indicated as the glutathione (GSH)/reactive oxygen species (ROS) ratio) and ΔΨm after treatment with sTRAIL:FeSOD. Human promyelocytic leukemia (HL-60) cells or erythroleukemia (K562) cells were treated with sTRAIL:FeSOD (1,000 ng/ml) for 0, 1, 2, 3, 4, 5 or 6 hours and then subjected to ROS, GSH and ΔΨm (JC-1) analysis as described above. **(D) **Analysis of mitochondrial cytochrome c release. Cells were also incubated for 6 hours with or without sTRAIL:FeSOD (1,000 ng/ml). Cytosolic (Cyto) and mitochondrial (Mito) fractions were prepared from these cells and assessed by Western blot analysis.

One parameter that is altered by the ROS production is mitochondrial membrane potential (ΔΨm), and cancer cells display a strong resistance to chemotherapeutic agents, which is probably due to their efficacious protection against the mitochondrial apoptotic pathway. To examine the role of the mitochondrial apoptotic pathway in K562 and HL-60 cells undergoing sTRAIL:FeSOD-induced apoptosis, ΔΨm was analyzed by flow cytometry. Figures 4A and 4B show an increase in rhodamine123 (Rh123) and JC-1 fluorescence intensity in all four cell lines except the normal T cells after treatment with sTRAIL:FeSOD, indicating that the mitochondria remained polarized during apoptosis in K562 and HL-60 cells. The lack of a significant change in Rh123 and JC-1 fluorescence intensity in normal T cells may be related to the stable redox status (Figures 3H and 3J). The data shown in Figure 4C imply that low levels of ROS and high levels of GSH maintain the ΔΨm and that hyperpolarized mitochondria can reduce ROS to a lower level. Cytochrome c release did not occur during sTRAIL:FeSOD-induced apoptosis (Figure 4D). LO2 are type II cells, and sTRAIL was able to apoptose the LO2 cells in the presence of mitochondrial dysfunction (Figures 1G, 4A and 4B).

### sTRAIL:FeSOD-induced death is dependent on caspase-8

**Figure 5:**
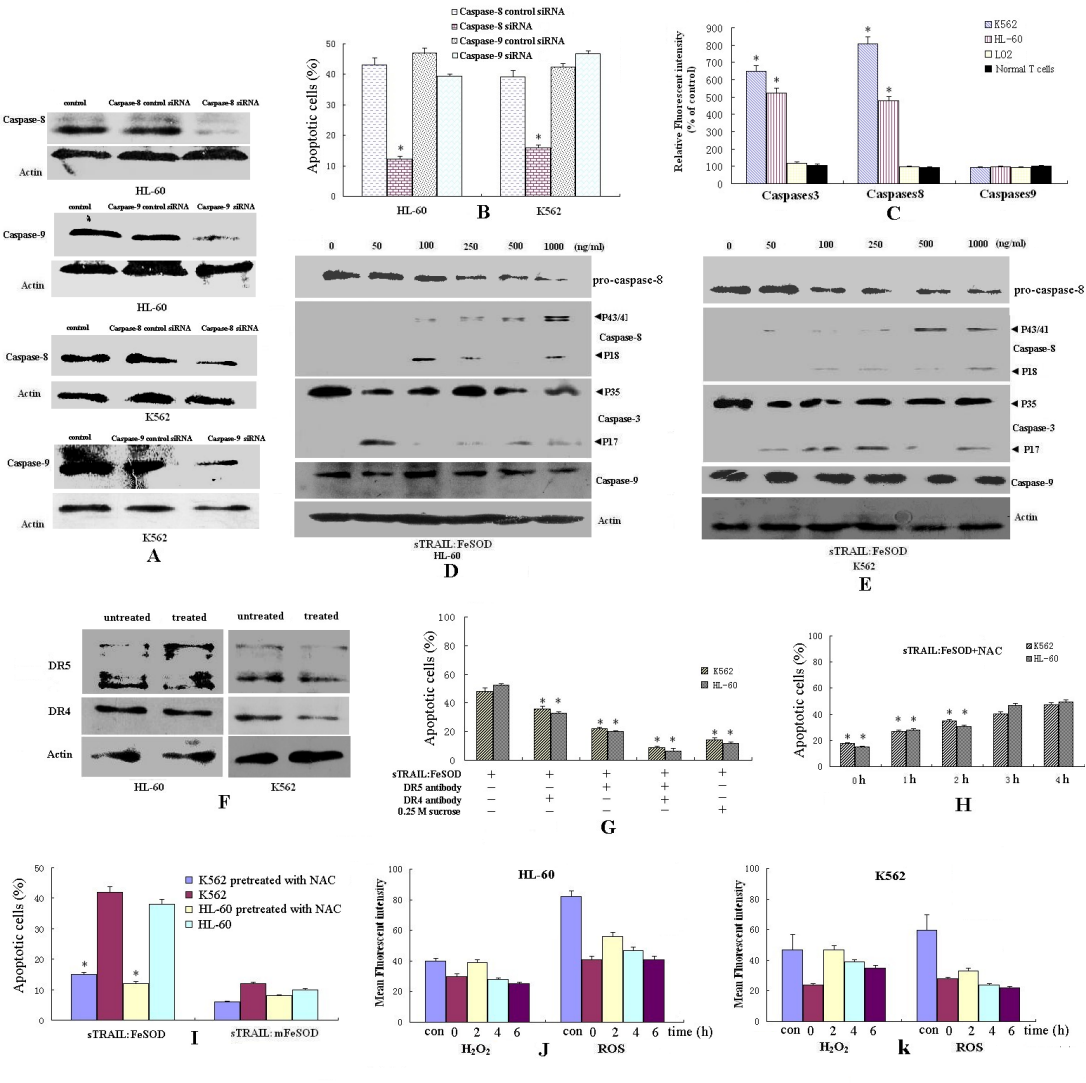
**Involvement of caspase-8 and the role of tumor necrosis factor-related apoptosis-inducing ligand (TRAIL) receptor and H**_**2**_**O**_**2 **_**in soluble forms of recombinant TRAIL:iron superoxide dismutase (sTRAIL:FeSOD)-induced apoptosis**. **(A) **and **(B) **Involvement of caspase-8 in sTRAIL:FeSOD-induced apoptosis. Caspase-8 or caspase-9 small interfering RNA was transfected into the indicated cells for 24 hours. After determining the inhibition of caspase-8 or caspase-9 expression **(A)**, cells were treated with sTRAIL:FeSOD (1,000 ng/ml) for 8 hours, and cell apoptosis was assayed by flow cytometry **(B)**. **(C) **Cells were treated with sTRAIL:FeSOD (1,000 ng/ml) for 6 hours. Cell lysates were tested for protease activity by the addition of caspase-specific peptides. Cleavage of the peptide by the caspase releases a chromophore, which was quantified using a fluorometer at 505 nm. **(D) **and **(E) **Cells were treated with sTRAIL:FeSOD at the indicated concentrations for 6 hours. Cell lysates were subjected to Western blot analysis using cleaved caspase-8, caspase-9 or caspase-3 antibody. **(F) **The DR5 and DR4 from untreated cells or cells treated with sTRAIL:FeSOD (for 6 hours) were quantified by Western blot analysis. **(G) **Cells were incubated with DR5 antibody and/or DR4 antibody for 1.5 hours prior to exposure to sTRAIL:FeSOD (1,000 ng/ml). **(H) **After cells were pretreated with sTRAIL:FeSOD for 0, 1, 2, 3 or 4 hours, 10 mM *N*-acetylcysteine (NAC) was added to the medium, and apoptosis was detected when cells had been treated with sTRAIL:FeSOD for 8 hours. **(I) **through **(K) **After pretreatment with 10 mM NAC for 24 hours, cells were incubated in fresh medium with 1,000 ng/ml sTRAIL:FeSOD or sTRAIL:mFeSOD for 8 hours. Apoptosis was determined by staining cells with anti-annexin V antibody and propidium iodide. H_2_O_2 _and reactive oxygen species levels were also detected after NAC-pretreated cells were treated with sTRAIL:FeSOD for 0, 2, 4 and 6 hours as described above. The untreated cells served as control. Data represent the mean ± SD of three independent experiments (*P < 0.05 vs. untreated control).

A common feature of cell death through apoptosis is the activation of caspases. The inhibition of caspase-8 expression effectively protected K562 and HL-60 cells from sTRAIL:FeSOD-induced apoptosis (Figures 5A and 5B). This result indicates that sTRAIL:FeSOD-induced apoptosis is dependent on caspase activation and that caspase-8 acts as the major initiator caspase in sTRAIL:FeSOD-induced apoptosis in these cells (Figures 5C to 5E). In contrast, decreased expression of caspase-9 did not significantly suppress sTRAIL:FeSOD-induced apoptosis in the leukemia cells (Figures 5A and 5B), suggesting the apoptosis to be caspase-9-independent. Changes in caspase-8 and caspase-3 activity in the two leukemia cell lines were markedly greater than those in the LO2 and T cells (Figure 5C), mirroring the different fate of the four cell lines after treatment with sTRAIL:FeSOD. In contrast to the control, there was no obvious change in caspase-9 activity in any of the four cell types.

### The internalization of FeSOD is indispensable for sTRAIL:FeSOD-induced apoptosis

First, we examined the expression of TRAIL receptors and whether treatment with sTRAIL:FeSOD led to elevated surface expression of DR4 and DR5. No major differences were found in the expression of either DR4 or DR5 after 6 hours of sTRAIL:FeSOD treatment (Figure 5F). To determine which receptor is involved in the death induced by sTRAIL:FeSOD and to discern whether the uptake of sTRAIL:FeSOD is receptor-dependent, we then performed an experiment in which we added blocking antibodies against DR5 and/or DR4 for 1.5 hours prior to sTRAIL:FeSOD exposure [29]. Figure 5G shows that blocking with DR5 or DR4 antibody partially protected the K562 and HL-60 cells from cell death, whereas the antibody against DR5 had a more pronounced protective effect on sTRAIL:FeSOD-induced apoptosis than did the antibody against DR4. No significant apoptosis was noted when blocking with both anti-TRAIL receptor antibodies. These results indicate that the sTRAIL:FeSOD-induced death signaling in the two cell lines depends on DR5 and DR4. After blocking with antibodies against DR5 and/or DR4, the internalization of the fusion protein mirrored the apoptosis results, showing death receptor dependence. There was minimal uptake of sTRAIL:FeSOD when pretreated with both anti-TRAIL receptor antibodies (data not shown), demonstrating the endocytosis to be receptor-mediated.

To provide evidence for the synergistic effect of sTRAIL and FeSOD, K562 and HL-60 cells were treated in hypertonic medium containing sTRAIL:FeSOD, conditions under which internalization of FeSOD was inhibited. Figure 5G shows that the sTRAIL:FeSOD-induced apoptosis drastically declined in the hypertonic medium, demonstrating FeSOD to be indispensable for sTRAIL:FeSOD-induced apoptosis. The confirmed internalization of sTRAIL:mFeSOD into K562 and HL-60 cells (Figure 2G) and the indistinguishable cell death in the two cell lines after treatment with sTRAIL:mFeSOD (Figures 1D and 1E) further prove the necessity for FeSOD to be internalized to allow for sTRAIL:FeSOD-induced apoptosis.

### A brief accumulation of H_2_O_2 _is involved in sTRAIL:FeSOD-induced apoptosis

We observed that the elevated H_2_O_2 _levels lasted about 2 hours in K562 and HL-60 cells after treatment with sTRAIL:FeSOD (Figures 3C and 3D). H_2_O_2 _has been reported to have a direct effect on caspase-8 activation, and therefore we investigated whether H_2_O_2 _was involved in sTRAIL:FeSOD-induced apoptosis. To that end, cells were treated with sTRAIL:FeSOD in the presence or absence of *N*-acetylcysteine (NAC) [30]. After pretreatment with sTRAIL:FeSOD for 0, 1 or 2 hours, the addition of NAC partially reduced sTRAIL:FeSOD-induced apoptosis (Figure 5H). When the cells were pretreated with sTRAIL:FeSOD for more than 3 hours, NAC did not show any significant inhibitory effect on sTRAIL:FeSOD-induced cell death. Thus, the results indicate that the production of H_2_O_2 _during O_2_^- ^scavenging contributed to sTRAIL:FeSOD-induced apoptosis and that caspase-8 activation by H_2_O_2 _occur within the first 2 hours after sTRAIL:FeSOD treatment. To further assess the role of H_2_O_2 _produced during O_2_^- ^scavenging in sTRAIL:FeSOD-induced apoptosis, we examined the apoptosis induced by sTRAIL:FeSOD after eliminating intracellular O_2_^- ^by pretreating HL-60 and K562 cells with NAC for 24 hours (Figures 5I to 5K). Because of the prescavenging of O_2_^-^, there was little substrate for FeSOD, which caused the H_2_O_2 _to remain at a very low level during the treatment with sTRAIL:FeSOD. Figure 5I shows that cell death was reduced. These data suggest that H_2_O_2 _plays an important role in sTRAIL:FeSOD-induced apoptosis.

### Downregulation of p-Akt and c-FLIP_L _promotes TRAIL-induced apoptosis in HL-60 and K562 cells

**Figure 6:**
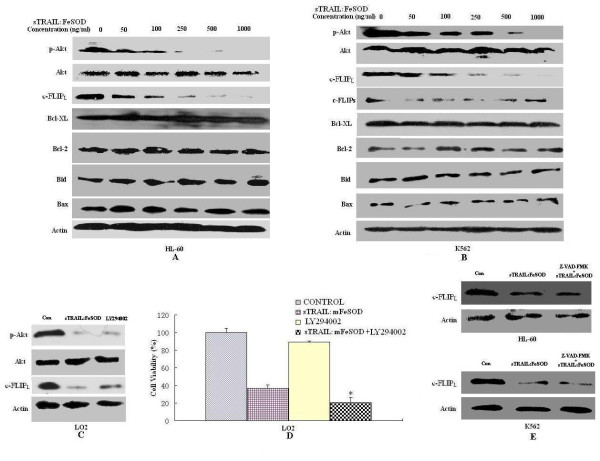
**Effects of soluble forms of recombinant tumor necrosis factor-related apoptosis-inducing ligand:iron superoxide dismutase (sTRAIL:FeSOD) on the levels of phosphorylated Akt and antiapoptotic proteins**. **(A) **and **(B) **Dose response of Akt dephosphorylation and cellular FLICE-inhibitory protein (c-FLIP_L_) downregulation by sTRAIL:FeSOD in human promyelocytic leukemia (HL-60) cells or erythroleukemia (K562) cells. Before they were disrupted, cells expressing phosphorylated Akt (p-Akt), c-FLIP_L _and c-FLIP_S _were treated with sTRAIL:FeSOD for 30 minutes, 1 hour and 1 hour, respectively. Bcl-xl, Bcl-2, Bax and Bid levels were detected after treatment with sTRAIL:FeSOD for 6 hours. **(C) **After treatment of LO2 cells with sTRAIL:FeSOD (500 μg/ml) or LY294002 (10 μM) for 4 hours, cell lysates were probed for p-Akt and c-FLIP_L_. **(D) **Enhancement of TRAIL-induced cytotoxicity caused by LY294002 (10 μM) in LO2 cells. Cells were pretreated with LY294002 for 30 minutes and then treated with sTRAIL:mFeSOD (1,000 ng/ml) or left untreated for 8 hours. Cell survival was determined by staining cells with anti-annexin V and propidium iodide. **(E) **In the presence or absence of the specific inhibitor of total caspase 50 μM (Z-VAD-FMK, benzyloxycarbonyl-Val-Ala-Asp (OMe) fluoromethylketone), cells were treated with 1,000 ng/ml sTRAIL:FeSOD for 1 hour, and then c-FLIP_L _levels were determined by Western blot analysis.

**Figure 7:**
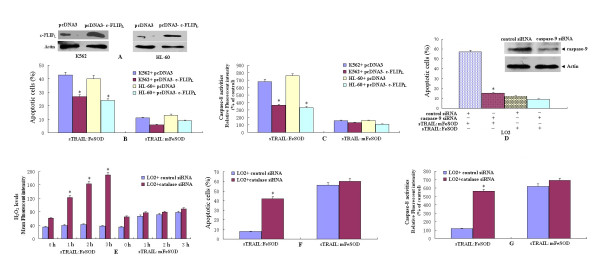
**Cellular FLICE-inhibitory protein (c-FLIP_L_) is involved in soluble forms of recombinant tumor necrosis factor-related apoptosis-inducing ligand:iron superoxide dismutase (sTRAIL:FeSOD)-induced apoptosis, and the lack of responsiveness to sTRAIL:FeSOD in LO2 cells results from low levels of H_2_O_2_**. **(A) **through **(C) **Effects of c-FLIP_L _overexpression on apoptosis and caspase-8 activities. After determination of c-FLIP_L _overexpression **(A)**, cells were treated with sTRAIL:FeSOD (1,000 ng/ml) or sTRAIL:mFeSOD (1,000 ng/ml), and apoptosis **(B) **and caspase-8 activity **(C) **were subsequently assayed. **(D) **Caspase-9 small interfering RNA (siRNA) was transfected into LO2 cells for 24 hours. After determining the inhibition of caspase-9 expression, cells were treated with sTRAIL:FeSOD (1,000 ng/ml) or sTRAIL:mFeSOD (1,000 ng/ml) for 8 hours, and cell apoptosis was assayed by flow cytometry. **(E) **through **(G) **Effect of H_2_O_2 _burst on cell death and caspase-8 activity in LO2 cells. After catalase siRNA transfection for 24 hours, cells were treated with 1,000 ng/ml sTRAIL:FeSOD for 0, 1, 2 or 3 hours, and then intracellular H_2_O_2 _was measured using dihydrorhodamine 123 **(E)**. Cells were treated with sTRAIL:FeSOD (1,000 ng/ml) or sTRAIL:mFeSOD (1,000 ng/ml), and apoptosis **(F) **and caspase-8 activity **(G) **were subsequently assayed. Data represent the mean ± SD of three independent experiments (*P < 0.05 vs. untreated control).

It is known that p-Akt can be activated by ROS and that elevated Akt activity protects cells against TRAIL-induced apoptosis. Figure 5H shows that decreased H_2_O_2 _levels did not entirely inhibit sTRAIL:FeSOD-induced apoptosis. Thus, we postulated that Akt may also be involved in sTRAIL:FeSOD-induced apoptosis in K562 and HL-60 cells. p-Akt was rapidly dephosphorylated within 30 minutes of adding sTRAIL:FeSOD without changing the Akt protein level, and sTRAIL:FeSOD treatment markedly depressed the c-FLIP_L _level in the two leukemia cell lines (Figures 6A and 6B). c-FLIP_S _was not detected in the HL-60 cells (Figure 6A). c-FLIP_S _was detected in K562 cells, but did not show any change in expression levels after sTRAIL:FeSOD addition (Figure 6B). Therefore, we hypothesize that c-FLIP_L _is involved in sTRAIL:FeSOD-induced apoptosis. sTRAIL:FeSOD-induced apoptosis in K562 and HL-60 cells was partially inhibited by c-FLIP_L _overexpression (Figures 7A to 7C), demonstrating that the p-Akt/c-FLIP_L _signaling pathway is also involved in sTRAIL:FeSOD-induced apoptosis. The effect of sTRAIL:FeSOD on the downregulation of c-FLIP_L _was also determined in cells in which total caspases were blocked (Figure 6E), further proving that c-FLIP_L _levels were modulated by Akt. Bcl-2 family proteins are important pro- and antiapoptotic proteins in the cells, but we were unable to detect any changes in Bcl-2, Bcl-X_L _and Bax levels or Bid cleavage after 6 hours of sTRAIL:FeSOD treatment.

### Low levels of H_2_O_2 _protect LO2 cells from sTRAIL:FeSOD-induced apoptosis

The downregulation of p-Akt and c-FLIP_L _was also observed in LO2 cells (Figure 6C), but not in normal T cells (data not shown). It was not immediately apparent why TRAIL sensitivity did not occur in LO2 cells. LO2 cells are type II, and TRAIL-induced apoptosis can be blocked by caspase-9 inhibition in these cells (Figure 7D). The results presented in Figure 3I and Figures 4A and 4B also suggest that sTRAIL-induced apoptosis in LO2 cells was dependent on mitochondria. Caspase-9 activity was low, and mitochondria remained polarized after treatment with sTRAIL:FeSOD, which inhibits the mitochondrial apoptotic pathway. Therefore, we hypothesized that the low levels of H_2_O_2 _prevented sTRAIL:FeSOD from converting LO2 into type I cells, which are characterized by high caspase-8 activity. To test this hypothesis, RNA interference transfection was performed to depress catalase expression in the LO2 cells, and the H_2_O_2 _levels were found to be much higher than those in the control group after treatment with sTRAIL:FeSOD (Figure 7E). High caspase-8 activity and apoptosis were induced by sTRAIL:FeSOD (Figures 7F and 7G) in the catalase-deficient LO2 cells. On the basis of these experimental data, LO2 cells are not converted into type I cells after treatment with sTRAIL:FeSOD. On the contrary, sTRAIL:FeSOD not only depressed caspase-9 activity but also maintained caspase-8 activity below a critical threshold because of the low H_2_O_2 _levels, rendering the LO2 cells insensitive to sTRAIL:FeSOD-induced apoptosis. Thus, without the accumulation of H_2_O_2_, downregulation of p-Akt and c-FLIP_L _was insufficient to activate enough caspase-8 to sensitize LO2 to TRAIL-induced apoptosis at a low level of oxidative stress.

## Discussion

Here we describe a novel therapeutic approach for treating cancer using the new cell-permeable fusion protein sTRAIL:FeSOD. The effects of sTRAIL and sTRAIL:FeSOD on apoptosis in LO2 cells in hyperosmotic medium did not show significant differences, suggesting that the FeSOD domain does not change the oligomerization state of sTRAIL (Figures 1B and 1C). When engaging the TRAIL receptors (TRAIL-R1 and TRAIL-R2), the trimeric sTRAIL:FeSOD triggered the TRAIL-induced apoptotic signaling pathway and simultaneously permeated the cell membrane via the receptor-mediated endocytic pathway. Movement throughout the cytoplasm provided FeSOD with the opportunity to scavenge O_2_^- ^originating from the mitochondria. Once sTRAIL:FeSOD engaged the TRAIL receptors, TRAIL-induced apoptosis signaling was triggered, seemingly prior to the elimination of intracellular O_2_^-^. However, in the current study, the brief accumulation of H_2_O_2 _and the downregulation of p-Akt occurred rapidly enough to enhance the sensitivity of K562 and HL-60 cancer cells to TRAIL-induced apoptosis. The internalization of sTRAIL:FeSOD was rapid (Figure 2) and triggered TRAIL-induced apoptosis signaling and the elimination of intracellular O_2_^- ^to occur nearly simultaneously. In addition, it has been suggested that Fas-associated protein with death domain and caspase-8 are not recruited soon enough because of the long delay in death-inducing signaling complex assembly in type II cells [31]. Thus, FeSOD had sufficient time to scavenge intracellular O_2_^- ^and then influence the course of TRAIL-induced apoptosis.

The inhibition of H_2_O_2 _accumulation or the overexpression of c-FLIP_L _partially suppressed sTRAIL:FeSOD-induced apoptosis, and thus we infer that H_2_O_2 _and p-Akt affect caspase-8 activity via different pathways. Constitutive phosphoinositide 3-kinase (PI3K)/Akt activity has been demonstrated to be one of the most effective antiapoptotic survival pathways in TRAIL-resistant cells, and therefore decreasing p-Akt levels is an important mechanism of averting TRAIL resistance. Tumor cells containing an activating somatic mutation in PI3K are relatively resistant to TRAIL-induced apoptosis [32,33]. After treatment with sTRAIL:FeSOD, p-Akt was downregulated in three cell lines (Figures 6A to 6C), depressing c-FLIP_L _expression [20]. However, the LO2 cells responded little to the fusion protein, in contrast to the synergistic apoptosis in TRAIL-resistant K562 and HL-60 cells [26]. The downregulation of Akt activity by LY294002 promoted TRAIL cytotoxicity in LO2 cells (Figure 6D), implying that low oxidative stress induced by FeSOD inhibited TRAIL-induced apoptosis in the LO2 cells. The results of the present study suggest that differences in caspase-8 activity determine the different cellular effects of sTRAIL:FeSOD on the indicated cells based on the interruption of the mitochondrial apoptotic pathway (Figures 4A, B and 5B to 5E). ROS have been reported to regulate caspase activation in TRAIL-resistant human colon carcinoma cells [17]. Perez-Cruz *et al. *[34] indicated that intracellular vitamin C can quench some of these ROS, reducing caspase-8 activation, similar to what we observed in the LO2 cells. However, Fas *et al. *[10] found that wogonin sensitizes resistant malignant cells to TRAIL by shifting levels of the TRAIL-induced free radical O_2_^-^, consistent with our results demonstrating the sensitization of K562 and HL-60 cells.

ROS levels have been found to be significantly higher in cancer cells than in normal cells, and the activity of antioxidant enzymes such as glutathione peroxidase, catalase and SOD has been shown to be significantly lower in cancer patients than in controls [35]. The higher ROS levels and lower antioxidant enzyme activities (Figures 3C to 3F) make the transient burst of H_2_O_2 _possible in K562 and HL-60 cells; however, because of the lower ROS levels and vigorous antioxidant system, the brief accumulation of H_2_O_2 _was not detected in LO2 cells (Figures 3G and 3I). The brief accumulation of H_2_O_2 _during O_2_^- ^scavenging was involved in the sTRAIL:FeSOD-induced apoptosis in K562 and HL-60 cells (Figures 5H to 5K), possibly exerting a direct effect on caspase-8 activation [36]. The low H_2_O_2 _levels protected LO2 cells from sTRAIL:FeSOD-induced apoptosis (Figures 7E to 7G). Normal T cells produced very little ROS, and therefore only a small shift in redox state was seen, which may explain why sTRAIL:FeSOD did not sensitize normal T cells to undergo apoptosis. Our studies reveal that sTRAIL:FeSOD reduces the level of intracellular O_2_^-^, with two results: the downregulation of p-Akt arising from a low level of O_2_^- ^and the transitory accumulation of H_2_O_2_, both of which may increase the amount of available active caspase-8 [37]. However, a low level of O_2_^-^, impairing caspase-8 activation [36], and stable mitochondria may inhibit apoptosis. Thus, we infer that the relative ratio of these opposing effects described above determines the sensitivity of a cell to sTRAIL:FeSOD and that this relative ratio may be associated with the cell type and the level of O_2_^-^. Without the accumulation of H_2_O_2_, the available activated caspase-8 was insufficient (Figures 5C and 7E to 7G). At the same time, caspase-9 activity was inhibited (Figures 4 and 5C), which means that the mitochondrial apoptotic pathway was interrupted. Thus, sTRAIL:FeSOD cannot sensitize LO2 cells to apoptosis when there is insufficient activated caspase-8 to excite the downstream apoptotic pathway in the absence of the intrinsic apoptotic pathway. The induction of apoptosis in K562 and HL-60 cells implies that the net result of intracellular O_2_^- ^scavenging by sTRAIL:FeSOD is the presence of a critical amount of activated caspase-8. Thus, in environments of low-level oxidative stress, the cellular context may influence the relative ratio of the opposing effects and subsequently determine whether this low level of oxidative stress favors apoptosis or survival.

Because of the effective protection against the mitochondrial apoptotic pathway mediated by the increased expression of Bcl-X_L _and the mutation of caspase-8, K562 and HL-60 cells exhibit strong resistance to chemotherapeutic agents [38,39]. However, after treatment with sTRAIL:FeSOD, K562 and HL-60 cells undergoing apoptosis retained their ΔΨm (Figure 4), and we also failed to detect a difference in the expression level of Bcl-2, Bax, Bid or Bcl-X_L _(Figures 6A and 6B) or a change in the distribution of cytochrome c (Figure 4D). The unchanged pattern of cytochrome c localization may be associated with a decrease in ROS levels, leading to mitochondrial stability. Depressed caspase-9 expression did not suppress apoptosis, demonstrating that sTRAIL:FeSOD-induced apoptosis was independent of the mitochondrial apoptotic pathway in K562 and HL-60 cells (Figures 5A and 5B). Mohr *et al. *[40] indicated that high levels of MnSOD protect colorectal cancer cells from TRAIL-induced apoptosis by inhibition of Smac/DIABLO release. However, this effect may be limited to type II cells, in which death receptor-mediated apoptosis is dependent on mitochondria. In contrast, in the present study, treatment with sTRAIL:FeSOD caused K562 and HL-60 cells to convert to type I cells and to apoptose independently of the intrinsic apoptotic pathway, consistent with the effects of erythroid differentiation [41]. Thus, mitochondrial hyperpolarization does not delay apoptosis when a critical amount of caspase-8 has been activated, which may be the mechanism that regulates type I cell apoptosis independently of the mitochondrial signaling pathway [36].

Death receptors are expressed in liver tissue as well as in isolated hepatocytes [5], and for this reason sTRAIL:FeSOD was able to permeate the cell membrane and reduce the oxidative stress in LO2 cells without causing cytotoxicity. Maintaining the balance of opposing effects is important when treating normal cells with sTRAIL:FeSOD, because an appropriate ROS level is extremely important for preserving vital cellular and biochemical functions. Our future work will focus on the task of estimating intracellular ROS production in an individual cell to determine a treatment dose that maintains ROS levels at an appropriate interval, killing cancer cells without inducing normal cell death. In light of the different sensitivities of different cell types to ROS and the hypersensitivity of tumor cells to decreased levels of ROS relative to normal cells, the ability to control TRAIL cytotoxicity through the regulation of intracellular ROS levels will be a breakthrough in the utilization of TRAIL. Undoubtedly, sTRAIL:FeSOD is a good potential therapeutic choice.

## Conclusions

In conclusion, our research has shown that a cell-permeable fusion protein, sTRAIL:FeSOD, selectively sensitized K562 and HL-60 cancer cells to TRAIL-induced apoptosis but did not sensitize normal human hepatocytes (LO2 cells) or T cells. sTRAIL:FeSOD is a potent antioxidant that produces H_2_O_2 _during intracellular O_2_^- ^scavenging and downregulates p-Akt and c-FLIP_L_. Thus, sTRAIL:FeSOD enhances TRAIL-induced apoptosis in leukemia cells and, at the same time, avoids the negative effects of high levels of ROS. After treatment with sTRAIL:FeSOD, the mitochondria were still intact, and the TRAIL-resistant K562 and HL-60 cells successfully converted into type I cells. In addition, the downregulation of c-FLIP_L _was not sufficient to sensitize LO2 to TRAIL-induced apoptosis without H_2_O_2 _accumulation. These data suggest that sTRAIL:FeSOD can serve as a promising pharmaceutical agent for the treatment of leukemia.

## Methods

### Reagents and antibodies

The fluorescent probes DHE, Rh123 and the peroxide-sensitive fluorophore DCFDA were purchased from Molecular Probes (Eugene, Oregon, USA). The total caspase inhibitor Z-VAD-FMK (benzyloxycarbonyl-Val-Ala-Asp (OMe) fluoromethylketone) and the Annexin V-FITC Apoptosis Detection Kit were obtained from Merck (Darmstadt, Germany). Caspase-9, caspase-3 and caspase-8 fluorometric assay kits were obtained from BioVision (Palo Alto, California, USA). Rabbit p-Akt (Thr308) antibody; mouse Akt antibody; rabbit c-FLIP_S _and c-FLIP_L _antibodies; Bcl-X_L_, Bcl-2, Bax and Bid antibodies; caspase-9, caspase-3 and caspase-8 antibodies; and horseradish peroxidase (HRP)-conjugated goat antirabbit antibody were purchased from Cell Signaling Technology (Boston, Massachusetts, USA), along with LY294002 (PI3K inhibitor). TRAIL, DR4 and DR5 antibodies and donkey antimouse immunoglobulin G-HRP were obtained from Santa Cruz Biotechnology (Santa Cruz, CA, USA). All of the remaining reagents were purchased from Sigma (Saint Louis, Missouri, USA).

### Cell lines and culture conditions

The pUCm-T-TRAIL vector was kindly provided by Pro Liu (Institute of Biochemistry and Cell Biology, Shanghai Institute for Biological Science, Chinese Academy of Science (Shanghai, China). *Escherichia coli *DH5α [*sup*E4 Δ*lac*U169 (80*lacZ*ΔM15) *hsd*R17 *rec*A1 *end*A1 *gyr*A96 *thi*-1 *rel*A1], *E. coli *BL21 (DE3) (*hsd*S *gal *(*λc*Its857 *ind*1 *Sam*7 *nin*5 *lac*UV5-T7 gene 1)), pET-28a(+) vector, pUCm-T vector and pET22-FeSOD vector were available at our institute. *E. coli *DH5α and *E. coli *BL21 (DE3) were maintained at 37°C in Luria-Bertani (LB) medium supplemented with the appropriate antibiotics. K562, HL-60 and LO2 cells were available in our laboratory and cultured in a humidified 5% CO_2 _atmosphere at 37°C in RPMI 1640 medium supplemented with 10% fetal calf serum and antibiotics (penicillin and streptomycin). Human peripheral T cells were prepared as described previously [42]. T cells were activated with 1 μg/ml polyhydroxyalkanoate for 20 hours, washed three times and then cultured for an additional 5 days in the presence of 25 U/ml interleukin 2. Transfections of K562, HL-60 and LO2 cells were performed with the expression vectors pcDNA3-c-FLIP_L_, caspase-8 and caspase-9 small interfering RNA (siRNA), catalase siRNA or control siRNA (Santa Cruz Biotechnology) using the Attractene transfection reagent (Qiagen, Hilden, Germany).

### Production, purification and immunoblot analyses of sTRAIL and sTRAIL:FeSOD

A DNA fragment encoding the extracellular domain of human TRAIL (sTRAIL; 114 to 281 aa) was amplified from the pUCm-T-TRAIL vector with the primers sTRAIL-*Nco*I (5'-CATGCCATGGTGAGAGAAAGAGGTCCTCAGAGAGTAG-3') and sTRAIL-*Sal*I (5'-ACGCGTCGACTCCGCCTCCACCGCCAACTAAAAAGGCC-3') and directionally inserted into pET28a(+) plasmid to form pET28-sTRAIL using the unique *Nco*I and *Sal*I restriction enzyme sites. The FeSOD fragment was amplified from the pET22-FeSOD vector, polymerase chain reaction-amplified with FeSOD-*Sal*I (5'-ACGCGTCGACTCATTTGTACAGCTCCCACTACCCT-3') and FeSOD-*Not*I (5'-ATAAGAATGCGGCCGCAGCTTTGGCCAAGTTTTC-3') primers and cloned in frame with the unique *Sal*I and *Not*I restriction enzymes, yielding plasmid pET28-sTRAIL:FeSOD. Nucleotide sequences of the insert regions within these constructs were independently verified by automated DNA sequencing.

*E. coli *BL-21 DE3 (pLys) were transformed with pET28-sTRAIL and then induced in the presence of 0.8 mM IPTG in 1 liter of LB culture medium at 27°C for 3 hours [43]. Bacterial pellets were then collected by centrifugation at 5,000 rpm for 5 minutes and washed twice with ice-cold PBS. Pellets were sonicated and lysed in Triton X-100 lysis buffer (10 mM TrisHCl, pH 7.6, 150 mM NaCl, 10% glycerol, 1% Triton X-100, 0.1 mM dithiothreitol (DTT), 0.1 mM phenylmethanesulfonylfluoride) for 30 minutes on ice. The lysis supernatant was then cleared by centrifugation and poured onto a column with 5 ml of nickel-nitrilotriacetic acid Superflow resin (Qiagen), and the packed resin was washed with buffer consisting of imidazole gradient concentrations (0 to 500 mM). The sTRAIL:FeSOD fusion protein was expressed at 37°C and lysed from pET28-sTRAIL:FeSOD as described above. The achieved sedimentation (sTRAIL:FeSOD fusion protein) was then dissolved in lysis buffer (10 mM Tris HCl, pH 7.6, 150 mM NaCl, 8 M urea) overnight. The lysate was purified as described above. After purification, the denatured protein was renatured in buffer (10 mM Tris HCl, pH 7.6, 150 mM NaCl, 0.5 M L-arginine, 0.5 mM GSH, 0.5 mM L-Glutathione oxidized, 10% glycerol, 10 μM Fe^3+^, 10 μM Zn^2+^) consisting of a gradient of urea concentrations (0 to 8 M) at 10°C. The renatured proteins were collected and flowed over a Sephadex G-100 column for gel filtration chromatography. By disordering the amino acid sequence in the conserved region (69 to 84 aa, 120 to 132 aa and 162 to 169 aa) of FeSOD, we also produced a mutant form of fused protein (sTRAIL:mFeSOD) that was deficient in its enzymatic activity. The expression of sTRAIL, sTRAIL:FeSOD and sTRAIL:mFeSOD was assessed by Western blot analysis. The TRAIL-induced apoptosis activity of sTRAIL, sTRAIL:FeSOD and sTRAIL:mFeSOD was assayed by treating the TRAIL-sensitive LO2 cells in hypertonic medium containing sucrose (0.25 M), conditions that inhibited the endocytosis of TRAIL, eliminating the effect of FeSOD [25]. The SOD activity of purified sTRAIL:FeSOD was determined using the xanthine/xanthine oxidase system. sTRAIL:mFeSOD was used as control to analyze the cellular effects of sTRAIL:FeSOD.

### Apoptosis assay

To identify early apoptotic changes, cells were seeded in six-well plates (5 × 10^5 ^cells/well) and incubated for 8 hours in the presence of FeSOD, sTRAIL, sTRAIL:FeSOD or sTRAIL:mFeSOD. Cells were collected at 1,000 rpm for 5 minutes at 4°C. After being washed, the cells were then stained with fluorescein isothiocyanate (FITC)-conjugated anti-annexin V antibody and PI, followed by flow cytometric analysis (Becton Dickinson, Franklin Lakes, New Jersey, USA). The results are presented as the percentage of apoptosis. For each sample, 10,000 events were acquired in each group. Experiments were performed three separate times for each cell line.

### Preparation of FITC-labeled sTRAIL:FeSOD conjugate

By labeling with FITC, we could trace the permeation process of sTRAIL:FeSOD through the cell membrane. sTRAIL:FeSOD (10 mg) was dissolved in 5 ml of 500 mM carbonate buffer (pH 8.5) without sodium azide. Ten milligrams of FITC were dissolved in 1 ml of anhydrous dimethyl sulfoxide immediately before use, and then 50 μl of FITC were added to the dissolved sample. The tube was wrapped in foil and then incubated and rotated at room temperature for 2 hours. The labeled sTRAIL:FeSOD was desalted with a Sephadex G-25 column to remove unreacted FITC. The fluorescence intensity of sample was measured by using a fluorescence spectrophotometer. FeSOD and sTRAIL:mFeSOD were treated as described above. No difference was observed in the efficiency of FITC-labeled sTRAIL:FeSOD compared with unlabeled protein in terms of inducing apoptosis in LO2 cells (data not shown).

### Determination of sTRAIL:FeSOD internalization by confocal microscopy and flow cytometry

Prepared K562 cells were washed extensively with PBS and incubated in RPMI 1640 medium containing 5 μg/ml FITC-conjugated sTRAIL:FeSOD for 5, 15 or 30 minutes at 37°C. After internalization, following three washes with PBS to remove unbound protein and an acid wash (0.2 M NaCl, 0.2 M acetic acid) to strip the surface-associated ligand, intracellular FITC was observed by LSCM (LSM510; Carl Zeiss, Jene, Germany), and quantitative data were analyzed by using flow cytometry. LO2 cells were incubated in RPMI 1640 medium containing hyperosmotic sucrose (0.25 M) [44] and 5 μg/ml FITC-conjugated sTRAIL:FeSOD and then counterstained with the DNA dye Hoechst 33342 (Sigma).

### Cellular oxidative stress assay by flow cytometry

After incubation with 1000 ng/ml sTRAIL:FeSOD in fresh culture medium for the indicated amount of time, cells were washed and incubated with 20 μM dihydrorhodamine 123 (DHR123) (for H_2_O_2_), 50 μM 2,3-Naphthalenedicarboxaldehyde (NDA) (for GSH), 20 μM DHE (for O_2_^-^) or 5 μM DCFDA (for total ROS) at 37°C for 30 minutes. The cells were then washed three times with probe-free phosphate buffer. The fluorescence intensity was measured by flow cytometry.

### Measurement of mitochondrial membrane potential

Cells were precultured in a 24-well plate at a concentration of 0.5 × 10^6 ^cells/well. Subsequently, cells were treated with 1,000 ng/ml sTRAIL:FeSOD, 500 ng/ml rTRAIL or 500 ng/ml FeSOD for 6 hours or were left untreated as controls. After treatment with sTRAIL:FeSOD, cells were harvested and washed with prewarmed PBS. The cells were then incubated with 10 μM Rh123 or 1 μg/ml JC-1 at 37°C for 30 minutes, washed and subsequently analyzed by flow cytometry.

### Caspase activity assay

Prepared cells (1 × 10^6^/ml) were treated for 6 hours with 1,000 ng/ml sTRAIL:FeSOD, 500 ng/ml rTRAIL or 500 ng/ml FeSOD and then lysed in ice-cold lysis buffer (Biovision) for 10 minutes. After centrifugation for 10 minutes at 15,000 × *g*, cell lysates were tested for protease activity by the addition of reaction buffer (containing 10 mM DTT) and caspase-specific peptides conjugated to the fluorescence AFC (7-amino-4-trifluoromethyl coumarin). Cleavage of the peptide by caspase releases the chromophore, which was quantified using a fluorometer at 505 nm.

### Western blot analysis

Treated cells were collected, washed in PBS and then lysed with lysis buffer on ice. Approximately 20 μg of lysed protein were separated by sodium dodecyl sulfate-PAGE and transferred to a nitrocellulose blotting membrane, blocked for 1.5 hours in blocking buffer (5% bovine serum albumin solution and 0.1% Tween 20 in Tris-buffered saline (TBST)). After three washes in TBST, membranes were probed with the indicated antibodies in blocking buffer overnight. After three washing steps in TBST, the blots were subjected to appropriate secondary antibodies for 1.5 hours in blocking buffer. After two washes in TBST for 30 minutes, proteins were visualized by chemiluminescence detection.

## Authors' contributions

HYT designed the research, performed the experiments and wrote the paper. YQ analyzed the data and performed part of the experiments. JYL performed part of the experiments. XGG designed the research and revised the paper. All authors read and approved the final manuscript.
